# Associations between light exposure and sleep timing and sleepiness while awake in a sample of UK adults in everyday life

**DOI:** 10.1073/pnas.2301608120

**Published:** 2023-10-09

**Authors:** Altug Didikoglu, Navid Mohammadian, Sheena Johnson, Martie van Tongeren, Paul Wright, Alexander J. Casson, Timothy M. Brown, Robert J. Lucas

**Affiliations:** ^a^Centre for Biological Timing, Division of Neuroscience, School of Biological Sciences, Faculty of Biology Medicine and Health, University of Manchester, Manchester M13 9PL, United Kingdom; ^b^Department of Neuroscience, Izmir Institute of Technology, Gulbahce, Izmir 35430, Turkey; ^c^Department of Electrical & Electronic Engineering, School of Engineering, Faculty of Science and Engineering, University of Manchester, Manchester M13 9PL, United Kingdom; ^d^Thomas Ashton Institute, People, Management and Organisation Division, Alliance Manchester Business School, Faculty of Humanities, University of Manchester, Manchester M13 9PL, United Kingdom; ^e^Thomas Ashton Institute, Centre for Occupational and Environmental Health, Division of Population Health, Health Services Research & Primary Care, School of Health Sciences, Faculty of Biology Medicine and Health, University of Manchester, Manchester M13 9PL, United Kingdom; ^f^Centre for Biological Timing, Division of Diabetes Endocrinology and Gastroenterology, School of Medical Sciences, Faculty of Biology Medicine and Health, University of Manchester, Manchester M13 9PL, United Kingdom

**Keywords:** melanopic, light, sleep, sleepiness

## Abstract

Experimental and interventional studies show that light can regulate sleep timing and sleepiness while awake by setting the phase of circadian rhythms and supporting alertness. The extent to which differences in light exposure explain variations in sleep and sleepiness within and between individuals in everyday life remains less clear. Here, we establish a method to address this deficit, incorporating an open-source wearable wrist-worn light logger (SpectraWear) and smartphone-based online data collection. We use it to simultaneously record longitudinal light exposure (in melanopic equivalent daylight illuminance), sleep timing, and subjective alertness over seven days in a convenience sample of 59 UK adults without externally imposed circadian challenge (e.g., shift work or jetlag). Participants reliably had strong daily rhythms in light exposure but frequently were exposed to less light during the daytime and more light in pre-bedtime and sleep episodes than recommended [T. M. Brown *et al.*, PLoS Biol. **20**, e3001571 (2022)]. Prior light exposure over several hours was associated with lower subjective sleepiness with, in particular, brighter light in the late sleep episode and after wake linked to reduced early morning sleepiness (sleep inertia). Higher pre-bedtime light exposure was associated with longer sleep onset latency. Early sleep timing was correlated with more reproducible and robust daily patterns of light exposure and higher daytime/lower night-time light exposure. Our study establishes a method for collecting longitudinal sleep and health/performance data in everyday life and provides evidence of associations between light exposure and important determinants of sleep health and performance.

## Significance

As quantitative targets for day- and night-time light exposure and avoidance start to appear, there is a need for descriptions of existing patterns of light exposure in human populations and their relationship with sleep and circadian rhythms. Our study represents an application of a light dosimeter measuring light in standardized metrics for circadian biology (melanopic irradiance) to a population in everyday life. We find widespread failure to meet recommended light exposure targets, especially in the evening and night, and significant associations between recent light exposure/avoidance and both daytime sleepiness and the timing of sleep. Our data support the potential for improving sleep timing and daytime alertness in the real world using light.

Light is the principal synchronizer of daily rhythms in behavior and physiology to external time (1) and has a direct impact on sleep propensity and sleepiness (2–4). Modern lifestyles impose deviations from natural patterns of light exposure (dimmer daytimes and, especially, brighter nights), with the potential to disrupt circadian rhythms and sleep, and impair performance, health, and well-being (5–16). There is therefore an urgent and ongoing need to determine the extent to which aberrant light exposure impacts sleep and circadian biology in human populations and to devise effective countermeasures.

While numerous carefully controlled experimental studies confirm the ability of light to affect human sleep and alertness (4, 17–23), the importance of these mechanisms in timing sleep and regulating subjective sleepiness in everyday life is less well established (24). To address this knowledge gap, it is necessary to collect data on light exposure and sleep and sleepiness in human populations carrying out their ordinary lives in longitudinal studies. Relationships between wake-time light exposure and sleep timing have indeed been reported (10, 12, 25–28). However, these datasets are far from complete. First, the potential for light to have a more acute impact on sleepiness has not been explored using this approach. Second, accurate information about light during the sleep episode is generally missing because personal wrist light loggers have typically been worn during sleep with the risk of occlusion by bedclothes. Finally, no longitudinal real-world measurements are available using melanopic equivalent daylight illuminance (EDI), which has recently been established as the standardized metric for light exposures in circadian and sleep studies (29, 30).

Here, we set out to determine personal profiles of melanopic EDI and explore associations with sleep and subjective alertness in everyday life. To achieve this, we established a technology suitable for large-scale data collection and incorporation into ordinary life, comprising a wrist-worn light logger (31) and a smartphone-based method for longitudinal data collection of sleep and alertness. We find that divergences from recommended patterns of light exposure are common even in our sample of people with limited exposure to circadian and sleep challenge (such as shift work and health problems); that sleep timing was correlated with patterns of light exposure; that reduced subjective sleepiness was associated with increased light exposure in the morning; and that increased sleep latency was associated with increased pre-bedtime light exposure.

## Results

### Light Exposure in Everyday Life.

**Fig. 1.**
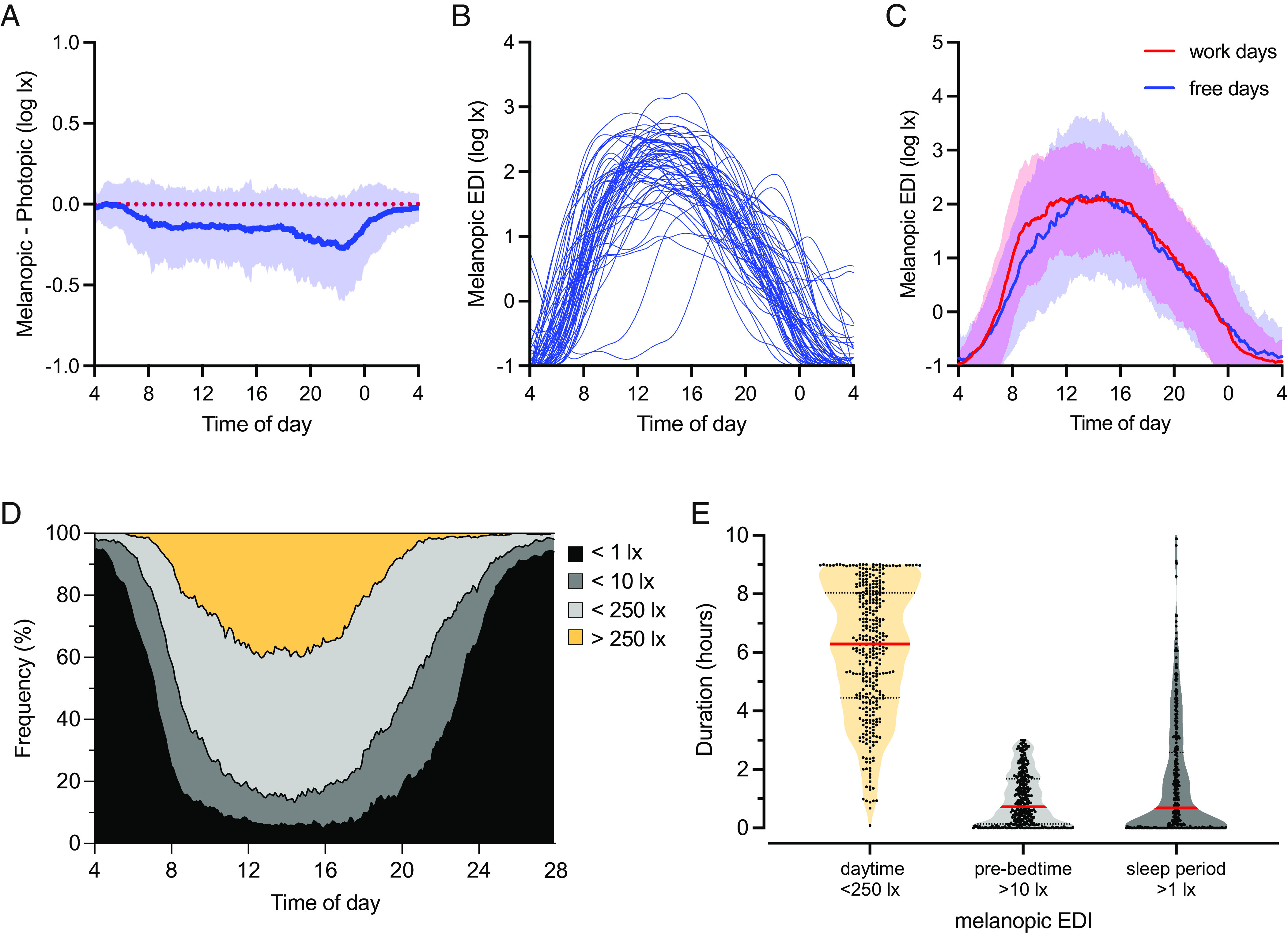
Light exposure in everyday life. (*A*) The difference between melanopic and photopic illuminances (log lx) across different times of day (clock time). The blue line shows the mean with SD. The red dotted line represents the expected relationship for ‘standard’ daylight. (*B*) Daily profiles of mean melanopic EDI (lx) as a function of clock time, depicted as a single smoothed line for each of 59 individuals. Individuals were represented by blue lowess smoothed fit lines. The *x* axis shows clock times. (*C*) Melanopic EDI (log lx) daily profiles (clock times) for workdays (red) and free days (blue). The lines show the mean with SD. (*D*) Frequency distribution of melanopic EDI as a function of time of day (0.1 h bins). The cumulative frequency of observations is colored according to the recent recommendations (33): melanopic EDI below 1 lx (black), between 1 and 10 lx (dark gray), between 10 and 250 lx (gray), and values above 250 lx (yellow). (*E*) Violin plots for exposure durations (hours) in three conditions: melanopic EDI below 250 lx in 8:00 to 17:00 daytime (yellow), above 10 lx in 3 h before bedtime (gray), and values above 1 lx during sleeping period (dark gray).

Light exposure patterns, alongside self-reports of longitudinal sleep and sleepiness states, were collected in 59 participants during their everyday life between February and July 2022 (sunrise ∼   between 5:00 and 7:30 and sunset ∼   between 17:30 and 21:30). We chose a wrist-worn format for the light logger because it was more ‘wear and forget’ than pendant or head-worn alternatives, while acknowledging that it would not provide a perfect record of light exposure at the eye and may sometimes be covered by clothing (31, 32). Participants were mostly younger than 35 y (86%, the maximum age group was 56 to 60 y) and female (66%). The study sample included participants who are students (49%) or full or part-time working (47%) (See *SI Appendix*, Table S1 for sample descriptions). The median study duration was 7.0 d (range: 0.8 to 11.5 d). Across the sample, there were strong positive correlations between all alpha-opic illuminances and photopic illuminance (r > 0.97, *P* < 0.001) (*SI Appendix*, Fig. S1*A*). Melanopic EDI was significantly lower than photopic illuminance (mean difference = −0.13, *P* < 0.001), indicating that on average, the light experienced by our participants was enriched for longer wavelengths compared to natural daylight. Moreover, this difference between melanopic and photopic illuminances was not equally apparent across all conditions. Rather, melanopic EDI was especially reduced for photopic illuminance <1,000 lx (*SI Appendix*, Fig. S1*B*) and between sunset and bedtime (Fig. 1*A*), consistent with reliance on electric light under these conditions. Individuals spent, on average, 14.7 h per day under melanopic EDI >1 lx and 1.7 h under very bright light (>1,000 lx melanopic EDI). The mean daily midpoint of this bright light exposure was 13:50 (range: 05:56 to 00:26), with the mean time of last bright light at 17:37. As expected, the timing of the last exposure to melanopic EDI >1 lx was significantly correlated with bedtime (r = 0.73, *P* < 0.001) consistent with the common practice of sleeping in darkened rooms. Light at night was very common and light exposure above a melanopic EDI of 10 lx was, at least partially, present for 41 participants with 0.2 h mean exposure between midnight and 04:00 (for 24 participants, exposure duration was above 30 min). Our participants thus had light exposure patterns consistent with stable alignment with the solar day, combined with substantial use of artificial light in the evening (*SI Appendix*, Fig. S1*C*). Most individuals experienced significant light exposure throughout the period between sunrise and bedtime (Fig. 1*B*), with only two participants showing substantially delayed phases of light exposure.

Participants reported their sleep and work schedules using a study-specific sleep diary. The average bedtime was 00:19 (SD = 2.0), and the average wake time was 08:22 (SD = 1.9). The study included 478 d of subjective diary reports (range: 4 to 11 d per participant). Among these, 58% of records were from workdays (this included studying days for students), 65% of which included commuting to a work/study place. On remaining ‘free’ days, the distribution of light exposure was wider compared to workdays, indicating more time spent in both bright (>1,000 lx) and very dim (<1 lx) conditions (*SI Appendix*, Fig. S1*D*). The mean bright light exposure duration per day was 1.4 h on workdays but 2.1 h on free days (*P* < 0.001). On workdays, people had earlier morning light exposure (10 to 100 melanopic lx) compared to free days (Fig. 1*C*).

Our study population experienced, on average, 3.6 h per day (range: 0.6 to 8.6) during which their wrist-measured light exposure was above the recently recommended (33) minimum daytime for corneal illuminance (melanopic EDI of 250 lx). This occurred mostly during the day (8:00 to 17:00; Fig. 1*D*), and the mean duration of daytime exposure to light above this threshold was 2.9 h (Fig. 1*E*). Nevertheless, light measures never reached this minimum recommended level on approximately 7% of days (Fig. 1*E*). Moreover, our data suggested that it was common for participants to exceed recommended limits for night and evening light (corneal melanopic EDI of <10 lx in 3 h before bed and <1lx during sleep). Thus, wrist-measured exposure >10 lx melanopic EDI in the 3 h before bedtime occurred on 89% of sampled days, with median exposure duration above this limit of 0.7 h (Fig. 1*E*). Furthermore, light in the sleep environment exceeded recommended corneal exposure limits on 76% of the 24 h epochs analyzed, with a median of 0.7 h under >1 lx melanopic EDI per sleep episode (Fig. 1*E*).

To explore other determinants of light exposure, we conducted linear regression between baseline-collected sociodemographic and lifestyle parameters (*SI Appendix*, Tables S1 and S2) and weekly light exposure characteristics (*SI Appendix*, Fig. S1*E*). This revealed that employed participants had more consistent daily patterns of light exposure (higher Interdaily stability; IS) and less light in the dimmest 5 h (lower L5). In addition, alcohol usage was associated with lower intradaily variation in light exposure (IV). Subjective evening chronotype had a near-significant association with a higher L5 but the midsleep time on free days corrected for sleep debt on workdays (MCTQ MSFsc) had a significant association. There were no associations between weekly light exposure and the Pittsburgh Sleep Quality Index (PSQI) score or social jetlag. Considering within and between individual variations, longitudinal mixed models (*SI Appendix*, Fig. S1*F*) indicated that workdays and having longer screen use duration were negatively associated with bright light (melanopic EDI > 1,000 lx) exposure. Smoking and alcohol usage were associated with above 1 lx melanopic EDI light during the sleep period.

### Relationships between Current Light Exposure and Sleepiness.

**Fig. 2.**
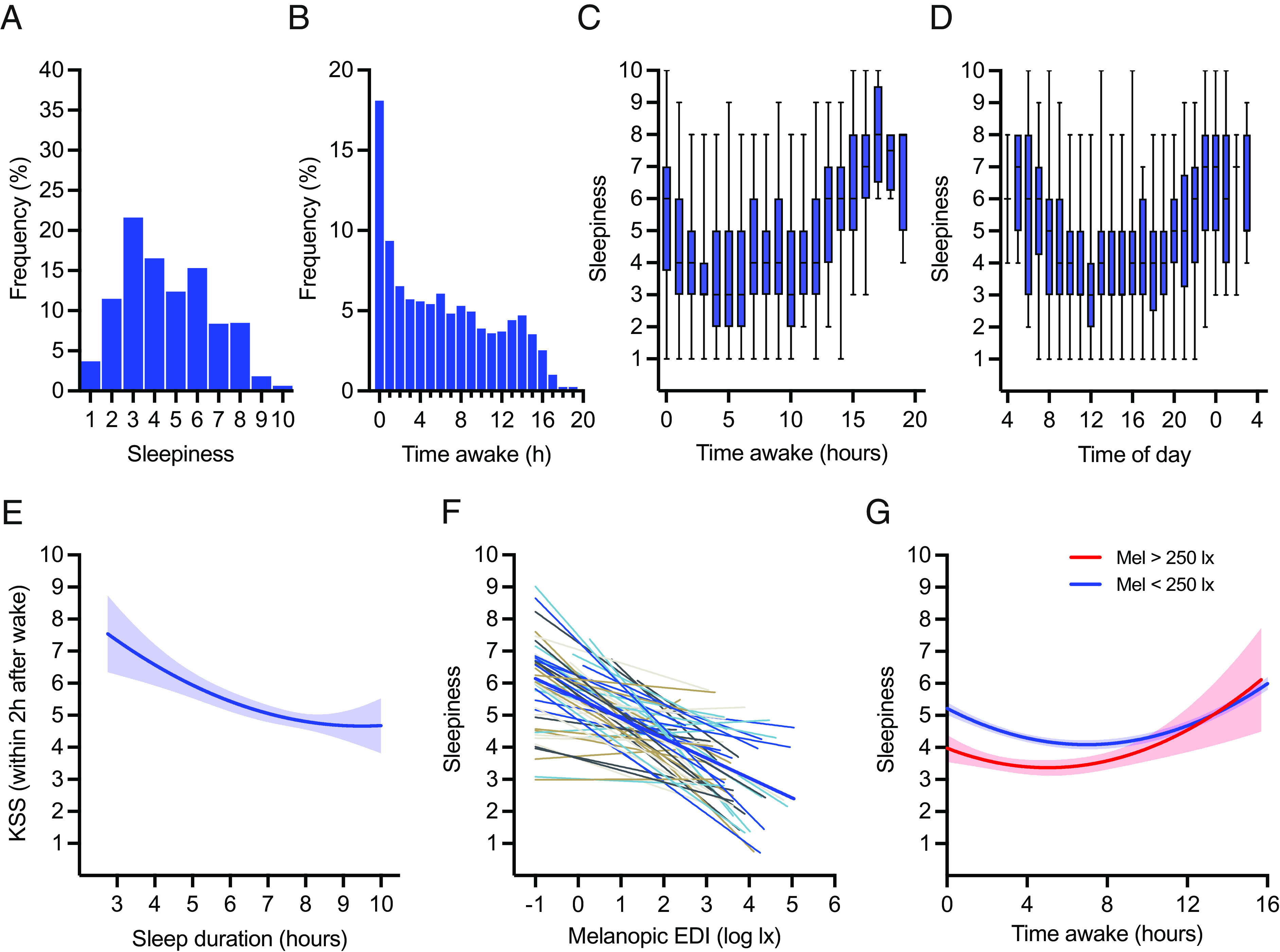
(*A*) Distribution of the sleepiness score collected using the Karolinska Sleepiness Scale (KSS), with 10 being extremely sleepy (1799 observations from 59 participants). (*B*) KSS distribution across time awake (hours). Time awake was calculated as the duration between KSS reporting time and wake time. (*C*) KSS in time awake (hours). The box plots show a median with an interquartile range (whiskers showing range). Quadratic fit line was estimated using a mixed model. Model: KSS = time awake + time awake^2^. The model estimates: intercept = 5.0, time awake term coefficient = −0.3 (*P* < 0.001), and time awake^2^ term coefficient = 0.02 (*P* < 0.001). (*D*) KSS across different times of day (clock time). The box plots show median with interquartile range (whiskers showing range). Harmonic fit line was estimated using a mixed model. Model: KSS = sine (2π × time of day/24) + cosine (2π × time of day/24). The model estimates: intercept = 5.0, sine term coefficient = 0.8 (*P* < 0.001), and cosine term coefficient = 1.1 (*P* < 0.001). The fit line: mesor = 5.0, amplitude = 1.4, and nadir = 15.5. (*E*) Association between awakening KSS and sleep duration (hours). The blue line shows quadratic fit line with 95% CI. Data analyzed by the quadratic mixed model. Model: KSS = sleep duration + sleep duration^2^. The model estimates: intercept = 11.2, sleep duration term coefficient = −1.5 (*P* = 0.001), and sleep duration^2^ term coefficient = 0.09 (*P* < 0.05). (*F*) Correlation between KSS and melanopic EDI (log lx) at the minute of KSS recording. Thin lines show the best-fit linear relationship for each participant. The thick blue line shows the population linear fit. Data were analyzed using a linear mixed model with random intercept and slope, melanopic EDI coefficient = −0.5 (*P* < 0.001). (*G*) KSS across time awake (hours) for melanopic EDI > 250 lx (red) and melanopic EDI < 250 lx (blue) subgroups. The lines show quadratic fit lines with 95% CI.

Participants scored their sleepiness on a scale of 1 to 10 throughout the day. We collected 1799 sleepiness records (range: 12 to 51 per participant). The mean sleepiness score was 4.6 (SD = 2.0) (Fig. 2*A*). The median clock time for KSS completion was 13:26 (1st qu. 09:38 to 3rd qu. 18:14), and the median times since wake was 5.8 h (1st qu. 1.8 h to 3rd qu. 10.9 h). Individuals were free to choose when to report sleepiness (while asked for at least three entries at different times per day), and sleepiness records were not evenly distributed across the wake episode, but rather most common in the first 2 h after awakening (Fig. 2*B*). During the 2-h post-wake period, sleepiness was higher than the sample average with a mean score of 5.1 (SD = 2.0) indicating a degree of sleep inertia (22) (Fig. 2*C*). We used diary entries to identify Karolinska Sleepiness Scale (KSS) records during the work/study period, these revealed generally lower sleepiness, with a mean KSS of 3.7 (SD = 1.4). As expected, sleepiness was higher again in the evening, with records after 20:00 having a mean of 5.7 (SD = 1.9) (Fig. 2*D*).

Analysis via longitudinal quadratic mixed model (*P* < 0.001) revealed that after the post-wake sleepiness (estimated KSS = 4.9) and then the midday alertness peak (at time awake 6.6 h), individuals accumulated sleepiness while awake (Fig. 2*C*). Further analysis via a longitudinal harmonic mixed model showed a daily rhythm in KSS score with peak alertness at 15:31 (*P* < 0.001) (Fig. 2*D*). Increasing sleep duration (up to 8.6 h) was associated with increasing awakening alertness of the subsequent wake episode (*P* < 0.001) (Fig. 2*E*).

Turning to associations with light exposure, across the full dataset, there was a negative association between current melanopic EDI and subjective sleepiness (*P* < 0.001) (Fig. 2*F*). This relationship survived adjustments for daily rhythm and duration since awakening (Table 1). There was high interindividual variation in this association (−1.6 to 0.8), but on average, a 1 log lx unit increase in current melanopic EDI was associated with a 0.5-point reduction in KSS. Time awake and current light level had a significant positive interaction (*P* = 0.01), indicating that sleepiness may specifically be related to light exposure in the mornings. Accordingly, the largest differences in KSS between occasions in which participants achieved or did not achieve the recommended 250lx melanopic EDI for daytime (33), occured over the first part of the wake episode (Fig. 2*G*).

**Table 1.** Relationship between current light exposure and sleepiness

	Standardized coefficient	SE	*P* value
**Melanopic EDI (log lx)**	−0.32	0.08	5.40e-05^***^
**Time awake (h)**	−0.70	0.16	1.38e-05^***^
**(Time awake)^2^**	0.70	0.12	8.90e-09^***^
**Sine(time of day)**	0.11	0.05	4.02e-02*
**Cosine(time of day)**	0.28	0.06	8.24e-06^***^
Time awake * Melanopic EDI	0.21	0.08	6.17e-03**
Sine(time of day) * Melanopic EDI	0.01	0.04	8.62e-01
Cosine(time of day) * Melanopic EDI	−0.11	0.06	7.25e-02

Mixed model with random participant intercepts and slopes was used to estimate sleepiness score collected using the 10-item Karolinska Sleepiness Scale (KSS), with 10 being extremely sleepy. Melanopic EDI (log lx) was the main predictor. The model was adjusted for a quadratic association with time awake (h) and harmonic association with time of day (radian). The model was also adjusted for daylength on the test day, if the test day is a workday, age, sex, baseline subjective health, smoking, alcohol and caffeine consumption on the test day. The sociodemographic, health, and season covariates were not significantly associated with the outcome. Number of observations: 1375, participant: 59. ^*^P < 0.05, ^**^P < 0.01, and ^***^P < 0.001.

### Relationships between Prior Light Exposure and Sleepiness.

**Fig. 3.**
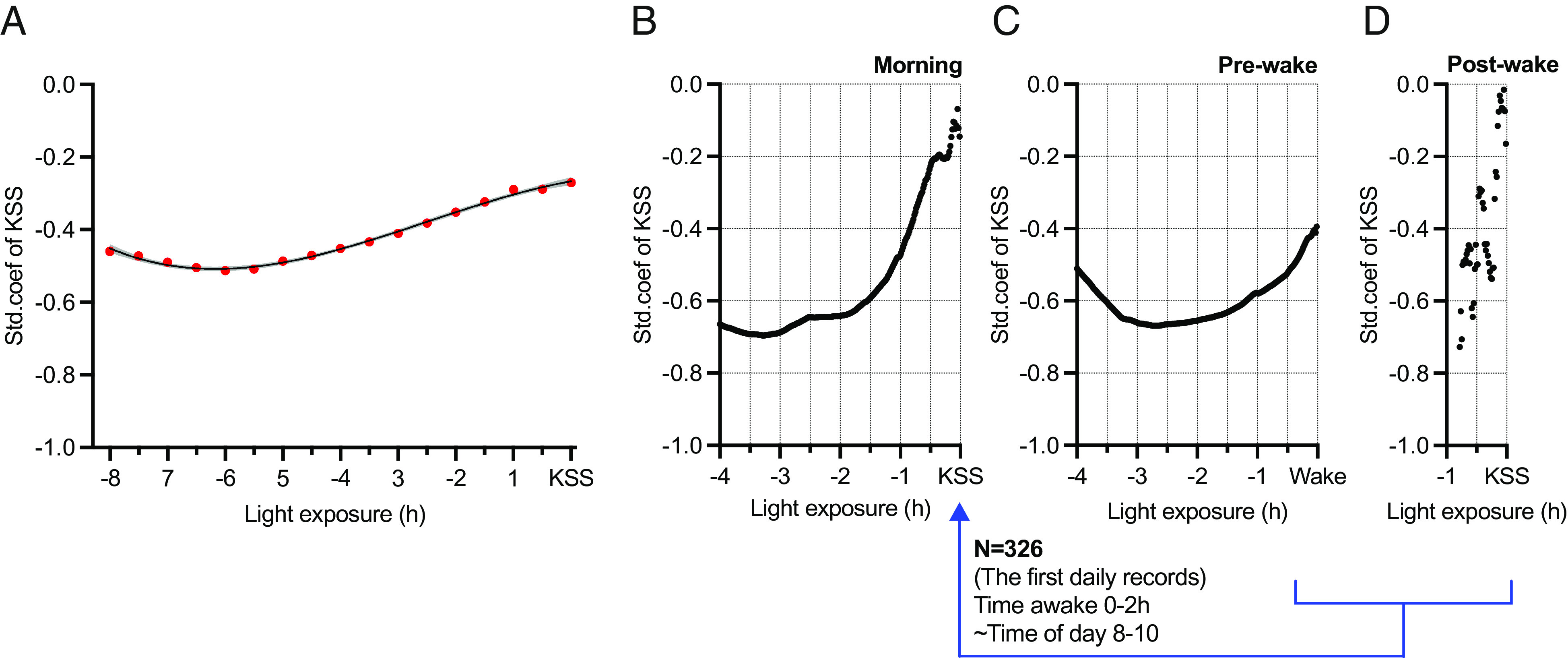
Recent history of light exposure and sleepiness and sleep latency. (*A*–*D*) The scatterplots show statistical model estimates for melanopic EDI (log lx) means across different exposure durations (hours). Each point is derived from a mixed model with random participant intercepts to estimate sleepiness score, collected using the 10-item Karolinska Sleepiness Scale (KSS). Melanopic EDI with different exposure durations were the main predictors. The models were adjusted for a quadratic association with time awake (h), harmonic association with time of day (radian), and their interactions with light exposure. The *x* axis shows light exposure duration (hours), while the *y* axis shows the standardized coefficient of KSS mixed models. (*A*) All KSS recordings were included in the models, and melanopic EDI histories (for every 30 min) were predictors (the line and CI show polynomial fit of standardized coefficient). The red color represents *P* < 0.05. In *B*–*D*, the predictors were melanopic EDI history (for every 1 min), and outcomes of the models were (*B*) the first KSS scores within 2 h after awakening, (*C*) the first KSS scores within 2 h after awakening, but the light exposure history was calculated starting from reported wake time instead KSS recording time, and (*D*) the first KSS scores within 2 h after awakening, but the light exposure history was calculated between the wake time and KSS recording time.

We next probed the timescales over which light exposure may correlate with sleepiness by assessing relationships between KSS and mean melanopic EDI (time awake and time of day adjusted) over different durations preceding record collection. The magnitude of the association between KSS and light exposure increased when mean melanopic EDI over the prior 6 h was used as the predictor (Fig. 3*A*). Considering that KSS was most frequently scored around wake time (Fig. 2*B*), one interesting implication of this finding is that the association with KSS might extend to light exposure prior to wake. To explore this possibility, we derived profiles of the strength of the relationship between KSS score and mean prior light exposure over different durations at 1-min resolution for records within the first 2 h after wake. In this reduced dataset, associations did not reach significance, but standardized coefficients between KSS and mean light exposure were negative throughout the prior 4 h (Fig. 3*B*). As this timeframe includes both pre-wake and post-wake times, we separated light exposure under these two conditions using data from the subject’s sleep log. These analyses indicated that light exposure both before and after wakening could contribute to any association between light and KSS in this post-waking period. Thus, relationships between KSS and light exposure were negative both over the last 3 h of sleep and after waking (Fig. 3 *C* and *D* respectively).

### Relationship between Daily Patterns of Light Exposure and Sleep.

**Fig. 4.**
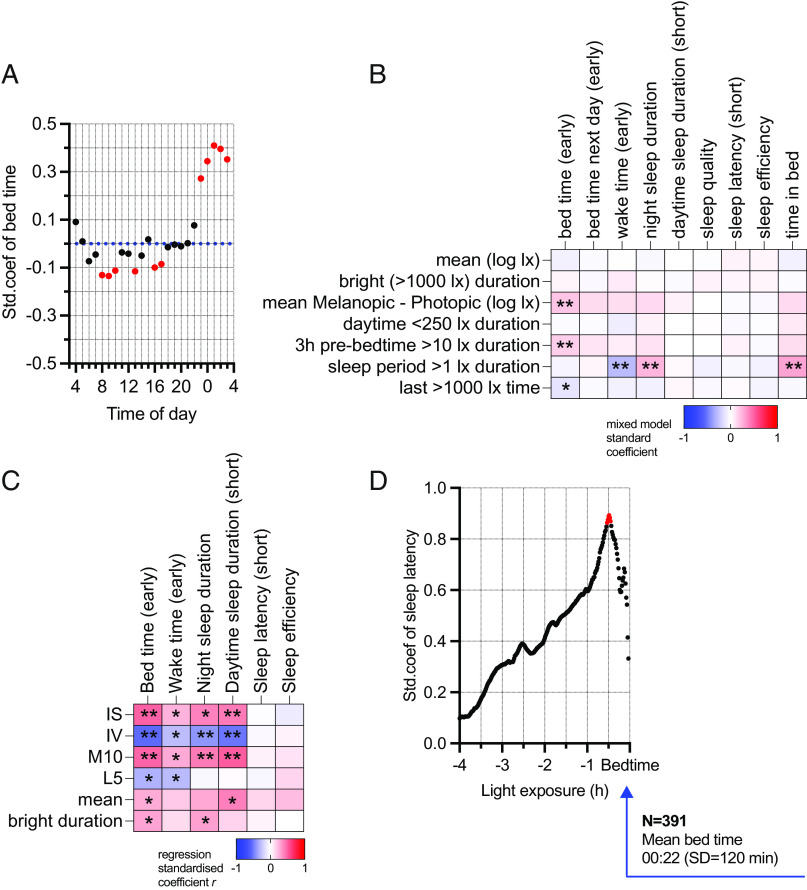
(*A*) Linear mixed models to compare hourly mean melanopic EDI (log lx) with bedtime on that day. The scatterplot shows statistical models for each hourly mean throughout the day. The *x* axis shows the time of day (clock time), while the *y* axis shows the standardized coefficient of the mixed models. The blue dotted line shows zero effect. The red color represents *P* < 0.05. (*B*) Linear mixed models to compare daily collected light exposure characteristics with daily sleep states. Mean: The mean of daily melanopic EDI (log lx). Bright duration: Total minutes with melanopic EDI more than 1,000 lx. Mean Mel - Pho: The mean of differences between all daily observations of melanopic EDI (log lx) and photopic illuminance (log lx). The next three columns show melanopic light exposure durations: daytime (8:00 to 17:00) below 250 lx, above 10 lx in the 3 h pre-bedtime, above 1 lx during sleeping period. Last time: The latest time with melanopic EDI more than 1,000 lx. These models were adjusted for daylength, work/rest status, smoking, and alcohol and caffeine consumption on the test day, as well as age, sex and baseline subjective health as covariates. The color scale represents standardized mixed model coefficients, with red being positive effect direction and blue being negative. Bedtime (clock time), wake time (clock time), night sleep duration (hours), daytime sleep duration (hours), subjective sleep quality (score 1 to 5), sleep latency (hours), sleep efficiency (%), and time in bed (hours) were collected using sleep diary data. Bedtime, wake time, daytime sleep duration, and sleep latency coefficients were inversed in the figure. *P < 0.05 and **P < 0.007. (*C*) Linear regression models to compare weekly light exposure characteristics with weekly sleep states. IS: Interdaily stability of melanopic EDI. IV: Intradaily variability of melanopic EDI. L5: The dimmest 5 h mean. M10: The brightest 10 h mean. Mean: The mean of weekly melanopic EDI (log lx). Bright duration: Average minutes with melanopic EDI more than 1,000 lx per day during the week. These models were adjusted for daylength on the test week, employment status, age, sex, baseline subjective health, baseline smoking, and baseline alcohol and caffeine consumptions. The color scale represents standardized regression coefficients where red is the positive effect direction and blue is negative. Bedtime (clock time), wake time (clock time), night sleep duration (hours), daytime sleep duration (hours), sleep latency (hours), and sleep efficiency (%) were calculated as the weekly average of sleep diary data. Bedtime, wake time, daytime sleep duration, and sleep latency coefficients were inversed in the figure. *P < 0.05 and **P < 0.008. (*D*) The scatterplot shows statistical mixed model estimates of sleep latency (hours) across melanopic EDI means with different exposure duration (hours). The models were adjusted for a linear association with time awake (h), linear association with bedtime (clock time), and their interactions with light exposure. The *x* axis shows light exposure duration (hours), while the *y* axis shows the standardized coefficient of mixed models (for every 1 min). The red color represents *P* < 0.05.

We next turned to potential relationships between light exposure and the timing, duration, and quality of sleep. Given the importance of appropriately timed light to set the circadian phase, we first investigated whether there was any association between light exposure at hourly intervals across the day and subsequent bedtime (Fig. 4*A*). The strongest correlations were between light exposure after midnight and late bedtime. These may be unsurprising given that people with earlier bedtimes would be sleeping at these times but are consistent with the potential for access to artificial light to reinforce late bedtimes. Similarly, the observed association between brighter morning light (8:00 to 10:00) and earlier bedtime might reflect late sleepers still being in bed at these times. However, there were associations between earlier bedtimes and light later in the day (between 13:00 and 17:00) when all participants were awake. Other day-to-day associations with sleep timing included habitual bedtime duration being longer and sleep occurring later and lasting longer on days on which sleep episodes had high >1 lx (Fig. 4*B*). Conversely, earlier sleep timing was observed on days with more natural light (smaller melanopic–photopic differential). More extensive exposure to light above the recommended limit of 10 lx melanopic EDI in the 3 h before sleep was also associated with early sleep time, possibly because the 3h before sleep more likely encompasses high daytime light exposure for early sleepers (Fig. 4*B*).

Our data also revealed associations in timing and duration of sleep and summary statistics for mean light exposure between individuals (Fig. 4*C*). Participants with more reproducible patterns of light exposure (high IS and low IV) and exposure to more light during the brightest 10 h of their day (M10) tended to have earlier bedtimes and less daytime sleep (Fig. 4*C*). Low IV and high M10 were also correlated with longer night-time sleep. Light metrics that were not designed to capture rhythmic patterns of exposure (daily mean light level and duration of bright (>1,000 lx melanopic EDI) light exposure) showed fewer substantial associations with sleep parameters (Fig. 4*C*).

We finally considered whether there was a relationship between light exposure in the period before bedtime and sleep latency. Light history analysis was performed as above for KSS, adjusted to include bedtime and duration of wake episode on that day in the models. Positive associations were returned, with a sharp peak for light exposure in the last 30 min before bedtime Fig. 4*D*. 1 log lx unit of light exposure was associated with longer sleep onset latency by 0.5 h.

## Discussion

There is abundant experimental evidence that light can regulate sleep and alertness in humans, but the extent to which sleep and alertness are affected by light in everyday life is less established. A first step to addressing that deficit is to collect data and describe relationships between light exposure and sleep/alertness during everyday life. This panel study provides a blueprint for achieving that objective. We used a wearable, open-source design, light dosimeter (31) constructed from modestly priced components to provide continuous recording of photoreceptor-specific, alpha-opic, irradiances (29) over several days. We paired this with a simple online self-reporting system via which participants could record a daily sleep diary and more frequent assessments of subjective sleepiness on their own smartphone device. This combination of a wearable light meter + smartphone-based data collection method allowed us to incorporate assessments of light exposure and sleep/sleepiness into participants’ everyday lives. Data collected with this method from our study population under everyday life contained associations between patterns of light exposure and both subjective sleepiness and the timing of sleep. Overall, regular and less-fragmented daily patterns of light exposure, and high-intensity daytime light and low-intensity nighttime light, were associated with early sleep/wake timings. In addition, recent light exposure was negatively correlated with morning sleepiness (‘sleep inertia’) and positively correlated with longer sleep onset latency at bedtime.

As recommendations for optimal light exposure patterns appear (33), there is an urgent need to describe existing patterns of light exposure in human populations. Wearing the light dosimeter as a wristband while awake and placing it at the bedside during sleep/rest enabled us to capture full-day information on melanopic EDI under real-world conditions (31). Although our method of measurement did not record corneal light exposure, our data suggest that people significantly diverge from recent recommendations (33). With one or two exceptions, our population had a primarily nocturnal sleep timing. Accordingly, most people achieved the recommended minimum of 250 lx melanopic EDI, even as measured at the wrist, for at least part of daytime (33). However, it was also very common for daytime light levels to fall far below this value, especially on workdays, indicating that indoor environments may frequently be too dim (27, 28). More widespread was divergence from the recommended exposures at evening and night-time, with many instances of readings >10 lx or >1 lx melanopic EDI in the pre-sleep and sleep periods, respectively (34). While the use of a wrist/bedside light dosimeter means that comparison with recommendations should be handled with care (35), these data do indicate that exposure to evening/nighttime light in particular is often rather high. Our data indicate that even in this population lacking common lifestyles causes of ‘circadian challenge’ (night shift work or jetlag), low daytime and high night-time light exposure are common. Such light exposure patterns are potential causes of circadian and sleep disruptions (1, 5–7, 33), with links to several physical and mental health disorders, cognitive dysfunction, occupational well-being and performance, educational attainments, and accidents (8–15).

Our data are consistent with published work suggesting that daily patterns of light exposure and light in prior days can affect the timing of sleep (while acknowledging that causation can work in the opposite direction). Wright and colleagues (1) showed that exposure to the natural light:dark cycle, characterized by stable continuous, bright daytime and no light at night, resulted in an earlier sleep timing. Similarly, analysis of UK Biobank data revealed that subjectively reported outdoor daylight exposure was linked to ease of getting up, fewer symptoms of insomnia, and early sleep timing preferences (9). Accordingly, earlier sleep times in our study were associated with stable, less fragmented, high in daytime and low in night-time light exposure. This finding is consistent with the ability of bright morning, and dim evening, light to enforce an advanced circadian phase (2). Accordingly, morning light can advance sleep phase (1, 13), while light in the evening and night can delay it (18, 36). Other correlations in our dataset (earlier sleep with higher light exposure in prior 3h; longer duration sleep with higher light exposure during sleep; and delayed sleep phase with night-time light) could be a simple reflection of when sleep occurs relative to the solar day. However, one less expected observation was the association between bright afternoon light exposure (until 17:00) and an earlier sleep timing. An exciting possibility is that this represents a real-world consequence of the ability of prior light exposure to modulate sensitivity to evening light exposure (37, 38).

Turning to subjective sleepiness, we found associations between recent light exposure and this parameter, especially in the morning sleep inertia period. Even in controlled experiments, the literature provides contradictory findings regarding the ability of light to influence subjective alertness in daytime. While there are sufficient data to conclude that light can have this effect (4, 20, 39–41), the fact that it is often not observed (42–45) confirms the ability of other influences on sleep (e.g., time awake and prior sleep) (23, 46) to preclude its detection. In principle, larger datasets such as that presented here have greater power to reveal associations with light in the face of such confounding factors. Indeed, our data also reveal an association between subjective sleepiness and prior sleep duration, while the association with light survives accounting for time of day and time awake. Our results thus suggest that light has an acute alerting effect, but this combines with longer-term effects of light and sleep history.

As lab-based research is clear about the alerting effects of light in the evening (17, 21, 38, 39, 42, 47, 48), it was surprising that we did not detect an association between evening light and subjective sleepiness. Two aspects of our study should be kept in mind when interpreting this finding. First, allowing ad libitum sleepiness reporting might have biased our dataset toward epochs when people are more alert and therefore remembered or felt like answering. Indeed, when the median sleepiness score per participant is accepted as zero, subjective sleepiness is slightly positively skewed indicating a more frequent alert record (*SI Appendix*, Fig. S2). Almost all participants have records of sleepy and alert conditions with enough range to test our hypotheses, but perhaps in the evening, we miss records of high sleepiness and low light exposure. Second, our application of a wrist-worn light monitor may underreport exposure to light from visual displays, which likely makes a strong contribution to light effects in the evening (36, 47).

An important question for light application in the real world is the timescale over which it exerts its biological effects. There is good evidence that the sort of non-image-forming light responses that could influence sleep and sleepiness can be defined by aggregated light exposure over a timeframe of tens of minutes to hours (48, 49). For sleepiness in everyday life, our data suggest associations with light exposure integrated over very long timescales, up to 6h. In the case of the strongest association (with morning sleep inertia), this implies an association with light both before and after waking. Previous experiments examining bright pre- or post-wake light were in line with this finding (22, 50, 51). The associations suggested here highlight the importance of continuous light logging both in defining the timescale of potential light effects and increasing the power to detect associations.

Another association suggested by continuous light logging was that between evening light exposure and sleep latency. We found that higher light exposure in the last 30 min before bedtime was associated with longer time to fall asleep after going to bed. This observation is consistent with experimental data showing that late light exposure not only delays the circadian phase but also impairs sleep latency, efficiency, and structure (3), and with another field study reporting an association between melanopic illuminance in the last 3h before bedtime and reduced sleep efficiency (34).

Our study represents a step in determining associations between light exposure and sleep/sleepiness in everyday life. We apply a dosimeter, available according to an open-access design, that provides the profiles of light exposure in melanopic EDI, the principal determinant of nonvisual effects of light. Combining with a smartphone method of data collection has revealed associations not only with daily sleep timing but also with subjective sleepiness while awake. Future work would benefit from a longer sampling duration (ideally at least 2 wk) and a larger and more diverse study population. Recent evidence shows large interindividual variation in light sensitivity (34, 52). Moreover, it is well established that light is not the only determinant of alertness or the timing or quality of sleep. Therefore, the next step in real-world light studies will be collecting larger datasets, both to improve statistical power and, perhaps more importantly, to identify the factors that determine when and to what extent light regulates sleep. The hope is that such an endeavor will lead to more personalized recommendations for adjusting light exposure to prevent or treat circadian disruption as well as other health and performance problems (53, 54). To this end, an open-access combined effort of longitudinal standardized measures is necessary (35, 55, 56). Here, we showed an example system consistent with everyday life, in which wearable light tracking using an open-access device in a wrist-worn form was combined with data collection of demographic and trait-of-interest information on an individual’s own personal smartphone. We adopted this strategy to explore associations with sleep and alertness, but it could be readily adapted to provide longitudinal data for other cognitive, behavioral, or physiological parameters.

## Materials and Methods

### Recruitment and Study Design.

Recruitment was carried out via local advertisements in the University of Manchester and word of mouth. Fifty-nine individuals participated in the study. The study aimed to include a diverse sample in terms of employment status, health, and sleep traits and therefore used minimal exclusion criteria: travel across time zones within 2 wk and age limit of 18 y. Participants (n = 4) reported undertaking ‘shiftwork’ on 7 d across the whole sample, but this was not associated with a substantial change in bedtime compared to the whole dataset (mean bedtime 00:48, wake time 08:37). We included these days of ‘self-reported’ shiftwork in analysis with the same motivation as our use of minimal exclusion criteria. Thus, we preferred our study group to approach a real-world population that could include subclinical and undiagnosed sleep and health problems (including potentially relevant conditions such as eye disorders) as well as extreme behavioral and occupational timing, while acknowledging that this may bring limitations to detect some associations. We aimed to collect data from minimum 50 participants to ensure the accuracy of the estimate in the longitudinal panel analyses (57). (See *SI Appendix*, Table S1 for sample descriptions). The data collection was performed between February and July 2022. The volunteers were paid a compensation for their time upon completion of the study period. This project was approved by the University of Manchester research ethics committee (Ref: 2021-12948-20856). Users confirmed informed consent through the online survey and provided written consent. Participants were requested to stay in the study for 1 wk, but they were free to stop at any time or extend the study period. During the study period, individuals were asked to 1) wear a light monitoring device, 2) complete four online baseline surveys in the registration meeting, 3) complete an online sleep and work diary once a day, and 4) report their subjective sleepiness multiple times throughout the day.

We used wearable light dosimeters, ‘SpectraWear’, comprising integrated multichannel light sensors, an ambient temperature sensor and accelerometer, which were manufactured in the University of Manchester (31) (*SI Appendix*, Fig. S3). The SpectraWear can measure photopic illuminance (lx) and α-opic equivalent daylight illuminances (EDI) (30), including melanopic EDI; it has ±15% accuracy under different illuminants in everyday life, can measure down to 0.1 lx (but with reduced accuracy below 1 lx), and has a near cosinusoidal angular response profile (half-width at half maximum sensitivity = 51°) (31). The monitor was set with a 30-s sampling interval. Participants were asked to wear the device during the day and to take it off just before their bedtimes by leaving it to a location in the same room (preferably near eye level). The device had up to 3 d of battery life; therefore, participants were told to charge the battery every day (preferably overnight). The light exposure measurements were started and stopped by a researcher in the registration and conclusion of face-to-face meetings; therefore, participants did not have control over the device or access to their measurements. In the registration meeting, users were requested to avoid clothing that occludes the light monitor.

During the registration face-to-face meeting, participants initially completed a baseline questionnaire. Then, they were introduced to two online surveys generated using Qualtrics software (Qualtrics, Provo, UT): 1) sleep and work diary and 2) sleepiness report. Participants were requested to complete the diary once per day (preferably directly after awakening) and to report their sleepiness at least three times throughout the day (preferably morning, middle of the day, and evening). They were explained that it was preferable to collect more data, at different times of the day, and that they should not limit themselves to three records. We emphasized that the aim was to measure regular, everyday life, behaviors, and they should not act differently nor try to change their sleep and lighting behaviors (see *SI Appendix*, Fig. S4 for an example study design for a participant).

### Surveys and Measures.

The baseline questionnaire battery included a study-specific sociodemographic and general health survey (*SI Appendix*, Table S2), the Pittsburgh Sleep Quality Index (PSQI) (58), the Munich Chronotype Questionnaire (MCTQ) (59), and the International Physical Activity Questionnaire (IPAQ) (60). To collect sleep and occupational schedules in the previous day, a study-specific diary was used (*SI Appendix*, Table S3). The study-specific surveys were based on the BrighterTime mobile app (61). The repeated measure of sleepiness (*SI Appendix*, Table S4) was collected by the Karolinska Sleepiness Scale (KSS) 10-item version (score 10: extremely sleepy) (62). In this repeated report throughout the day, participants were also asked to write their daytime sleeping occasions. During the study, the median time that participants spent in online surveys (diary or KSS) was 34 min.

The sociodemographic and health survey consisted of age, sex, employment status, subjective health rating (five-item from very bad to very good), subjective chronotype (four-item from definitely morning type to definitely evening type), previous diagnosis (sleep, eye, mental, and neurological disorders), smoking (no/yes, including vaping and nicotine supplements), general daily caffeine (units), and alcohol (no/yes) intakes. Using the MCTQ, social jetlag (hours) was calculated as the difference between midsleep in free days and workdays (63). As a measure of chronotype, MSFsc was also calculated as the midsleep time on free days corrected for sleep debt on workdays (64). Individuals’ physical activity levels were categorized as high, moderate, or low according to their IPAQ scores (65). As a measure of sleep quality, the PSQI score (out of 21 with a higher score indicating sleep problems) was used (58).

The sleep diary assessed bedtime, wake time, time to commute to work/study places, and time to return home from workplaces. Participants reported their sleep duration (hours) and sleep latency (minutes, how long it took to fall asleep after going to bed). The time in bed (hours) was calculated as the duration between wake time and bedtime. The sleep efficiency was calculated as the ratio of sleep duration to total duration in bed (%). In the diary, participants also reported their alcohol (units), smoke (no/yes), and caffeine (units) consumption yesterday as well as their napping (no/yes), outdoor daylight exposure duration (>4 h or not), screen duration (>4 h or not), and where they worked (home or not).

### Statistical Analysis.

All data cleaning, analyses, and graphing were performed using R version 4.0.4 (2021) and GraphPad Prism version 9.4.1 (2022). The raw sensor data were collected by 13 SpectraWear devices and converted to α-opic EDI (lx) and photopic illuminance (lx) using the device-specific calibration coefficients (31). In descriptive summaries and statistical analyses, log_10_ normalized lux values were used (see *SI Appendix*, Fig. S4 for example continuous melanopic EDI data of a participant). Interdaily stability of melanopic EDI (IS) was calculated as a metric of similarity of daily light exposure patterns during the study period by comparing the variance of average hourly means to the variance of all hourly means (66). Intradaily variability of melanopic EDI (IV) was calculated to investigate the fragmentation in light exposure patterns during the study period by comparing the mean square deviation of hourly means from the previous hour to the variance of all hourly means (66). We calculated the mean melanopic EDI, the mean melanopic EDI of the dimmest 5-h period (L5), the mean melanopic EDI of the brightest 10-h period (M10), and the total duration (minutes) with bright melanopic EDI (>1,000 lx). We also calculated daily light exposure variables: the mean of daily melanopic EDI (days were accepted to start at 4:00), bright duration of total minutes with melanopic EDI more than 1,000 lx, the last time with melanopic EDI more than 1,000 lx, the mean of differences between all daily observations of melanopic EDI (log lx) and photopic illuminance (log lx), and melanopic light exposure durations in daytime (8:00 to 17:00) below 250 lx, in above 10 lx in the 3 h pre-bedtime, in above 1 lx during sleeping period.

Our analyses were divided into four sections. In the first part, light exposure patterns in everyday life were described using density plots and time-of-day distributions. Associations between health, behaviors, employment, sociodemographic cofactors, and light exposure were investigated using linear regression models for grand averages of participants and linear mixed models for daily variables. These and the following linear mixed models (LMMs) were computed using the lme4 (1.1 to 27.1), lmerTest (3.1 to 3), and lm.beta (1.6 to 2) packages in R and composed of individuals as a random effect. Statistical results were presented with p-value significance limit of 0.05 and its adjusted versions by dividing it to the number of multiple tests with different predictors.

In the second part, we described KSS with histograms and distributions across different times of day (clock time) and time awake values (time between sleep diary-collected wake time on that day and KSS recording time). Daily rhythmicity of KSS was tested using LMM with a harmonic fit [sine (2π × time of day/24) + cosine (2π × time of day/24)]. Association between time awake and KSS was tested using LMM with a quadratic fit [time awake + time awake^2^]. Light observations within 1 min when the KSS was recorded were accepted as acute exposure. Immediate effects of melanopic EDI on KSS were assessed using the LMM adjusted for the harmonic time of day fit and the quadratic time awake fit as well as their interaction terms with light exposure. The model was adjusted for daylength, work/rest status, smoking, and alcohol and caffeine consumption on the test day, as well as age, sex and baseline subjective health as covariates.

The third section was built up on the previous multivariate acute LMM to investigate the impact of light history. We calculated the light history as the mean log-normalized melanopic EDI for a target duration preceding the observations. For example, the time zero light exposure corresponds to light at the time of KSS completion and 30-min light history to the mean of the previous 30 min. The same LMM model in the second part was used, but instead of acute light exposure, we used light history. We performed the LMM models for each 30 min of light histories up to 8 h and compared standardized coefficients. To assess the direction of the effect of light history with a high resolution, associations between light exposure and KSS were graphed across different light exposure durations (for every minute up to 4 h) in pre-wake times and post-wake times. The next section also tested sleep latency with a similar method as an outcome in addition to KSS but adjusted for a linear relationship with time awake and bedtime (instead of time of day).

The final section included comparisons of hourly light exposure means for each day and daily bedtimes using linear mixed models. This section also included comparisons of weekly light exposure variables and sleep states using linear regression models (adjusted for daylength on the test week, employment status, age, sex, baseline subjective health, baseline smoking, baseline alcohol and caffeine consumptions) for grand average of participants and within-individual comparisons of daily light exposure variables and sleep states using linear mixed models (adjusted for daylength on the test day, if the test day is a workday, age, sex, baseline subjective health, smoking, alcohol and caffeine consumptions on the test day).

## Supplementary Material

Appendix 01 (PDF)

## Data Availability

Software and hardware designs of the wearable light dosimeter data have been deposited in GitHub (10.48420/21946469) (31). Anonymized data of full light exposure and sleepiness measurements data have been deposited in Figshare (10.48420/23786238) (67).
