# Supplementary material for: Associations between light exposure and sleep timing and sleepiness while awake in a sample of UK adults in everyday life
Source: Proc Natl Acad Sci U S A. 2023 Oct 9;120(42):e2301608120. doi: 10.1073/pnas.2301608120 (PMC10589638; doi:10.1073/pnas.2301608120)
Supplement: Supplementary file 1 — Appendix 01 (PDF) [file pnas.2301608120.sapp.pdf]

Associations between light exposure and sleep timing and sleepiness while awake in a sample of UK adults in everyday life

Supplementary materials

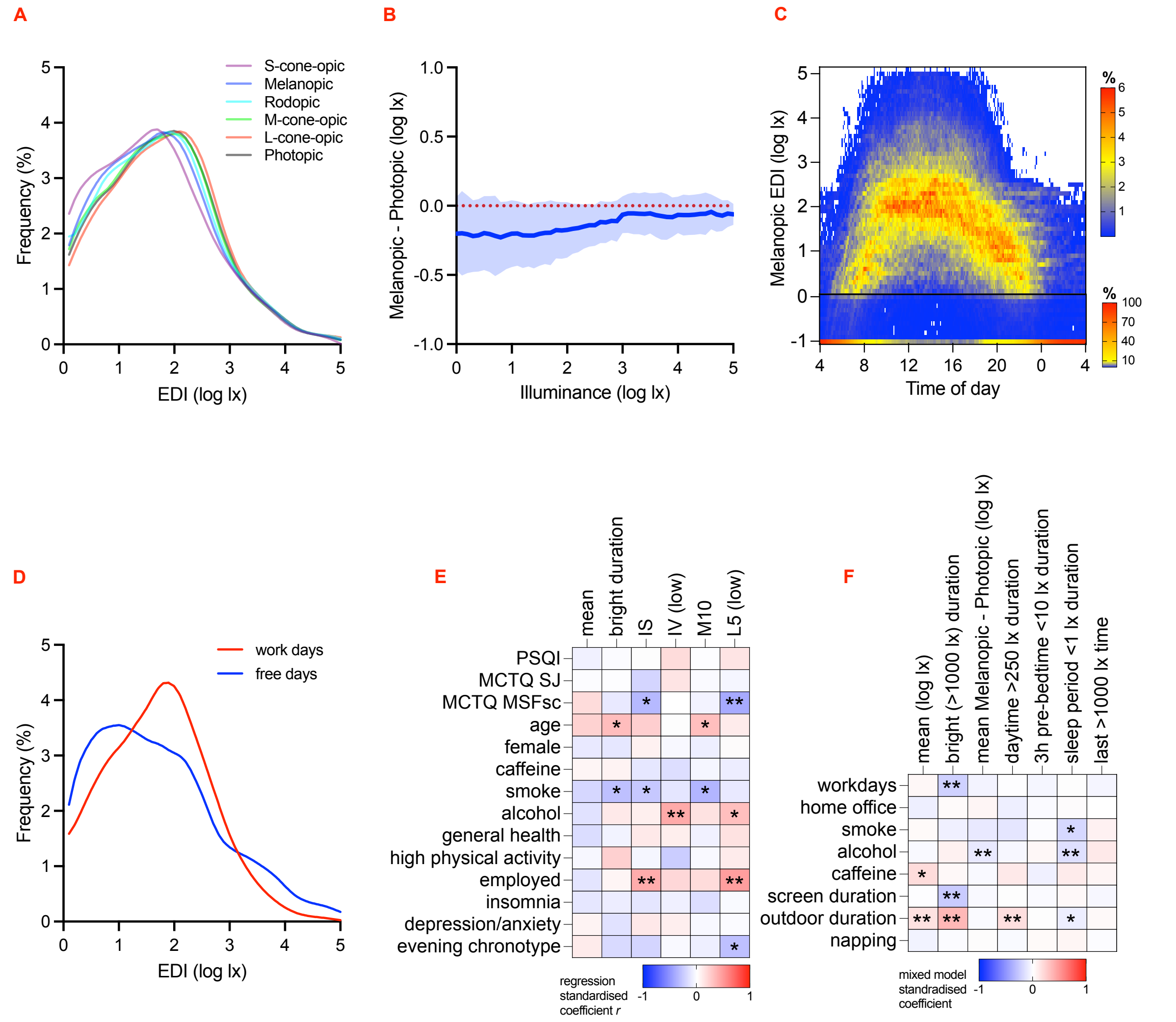

Figure S1: A) Distribution of α-opic equivalent daylight illuminance (EDI)s and photopic illuminance (log lx) between 1 to 10,000 lx. **B)** The difference between melanopic and photopic illuminances (log lx) in increasing illuminance values between 1 to 10,000 lx. The blue line shows the mean with standard deviation. The red dotted line represents the expected relationship for 'standard' daylight. **C)** Frequency distribution of melanopic EDI as a function of time of day (0.1h bins). Note that the frequency of observations is pseudocoloured separately for melanopic EDI between 1 and 10,000 lx (0-6% colour scale) and values lower than 1 lx (0-100%). **D)** The distribution of melanopic EDI (log lx) for workdays (red) and free days (blue) between 1 to 10,000 lx. **E)** Linear regression models to compare baseline-collected sociodemographic, sleep and health cofactors with weekly light exposure characteristics. MCTQ SJ: Munich Chronotype Questionnaire social jetlag (hours). MCTQ MSFsc: Munich Chronotype Questionnaire Mid-sleep time on free days corrected for sleep debt on workdays (clock time). PSQI: Pittsburgh Sleep Quality Index (score 0-21). IS: Interdaily stability of melanopic EDI. IV: Intradaily variability of melanopic EDI. L5: The dimmest 5 hours mean. M10: The brightest 10 hours mean. Mean: The mean of weekly melanopic EDI (log lx). Bright duration: Average minutes with melanopic EDI more than 1000 lx per day during the week. The colour scale represents standardised regression coefficients with red being positive effect direction and blue being negative. IV and LS coefficients were inversed in the figure. * p<0.05, ** p<0.0035. **F)** Linear mixed models to compare daily collected cofactors with daily light exposure characteristics. Mean: The mean of weekly melanopic EDI (log lx). Bright duration: Total minutes with melanopic EDI more than 1000 lx. Mean Mel - Pho: The mean of differences between all daily observations of melanopic EDI (log lx) and photopic illuminance (log lx). The next three columns show melanopic light exposure durations: daytime (8am-5pm) above 250 lx, below 10 lx in the 3h pre-bedtime, below 1 lx during sleeping period. The last column is the latest time with melanopic EDI more than 1000 lx. The colour scale represents standardised mixed model coefficients where red being positive effect direction and blue being negative. * p<0.05, ** p<0.0062

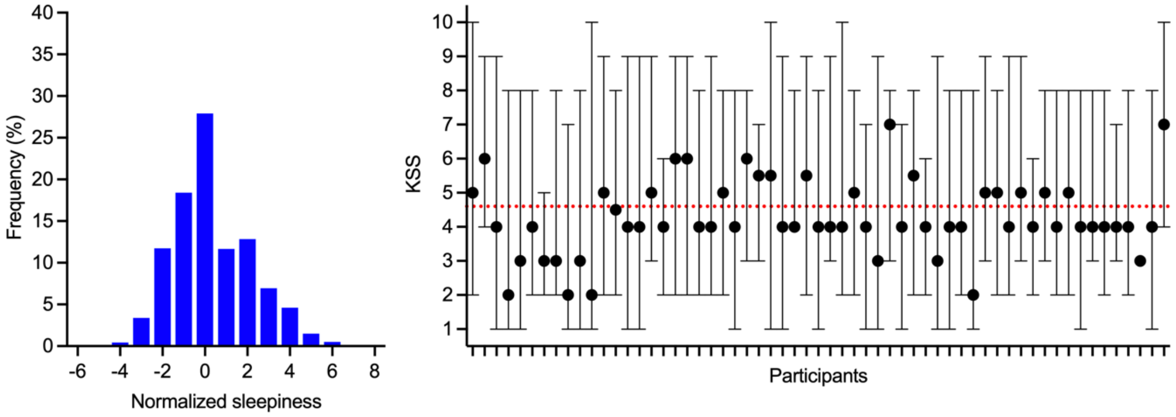

Figure S2: A) Distribution of normalized Karolinska Sleepiness Scale (KSS) with 10 being extremely sleepy (1799 observations from 59 participants). The scores were normalized to the median score of each participant. **B)** Median KSS score for each participant (error bars show the range). The dotted red line shows the sample mean.

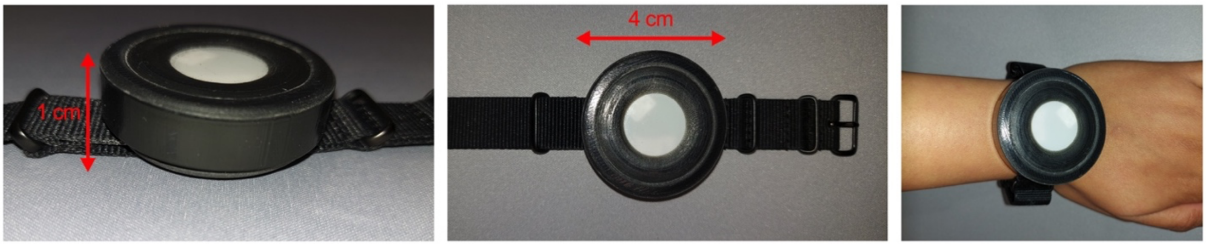

Figure S3: SpectraWear light dosimeter

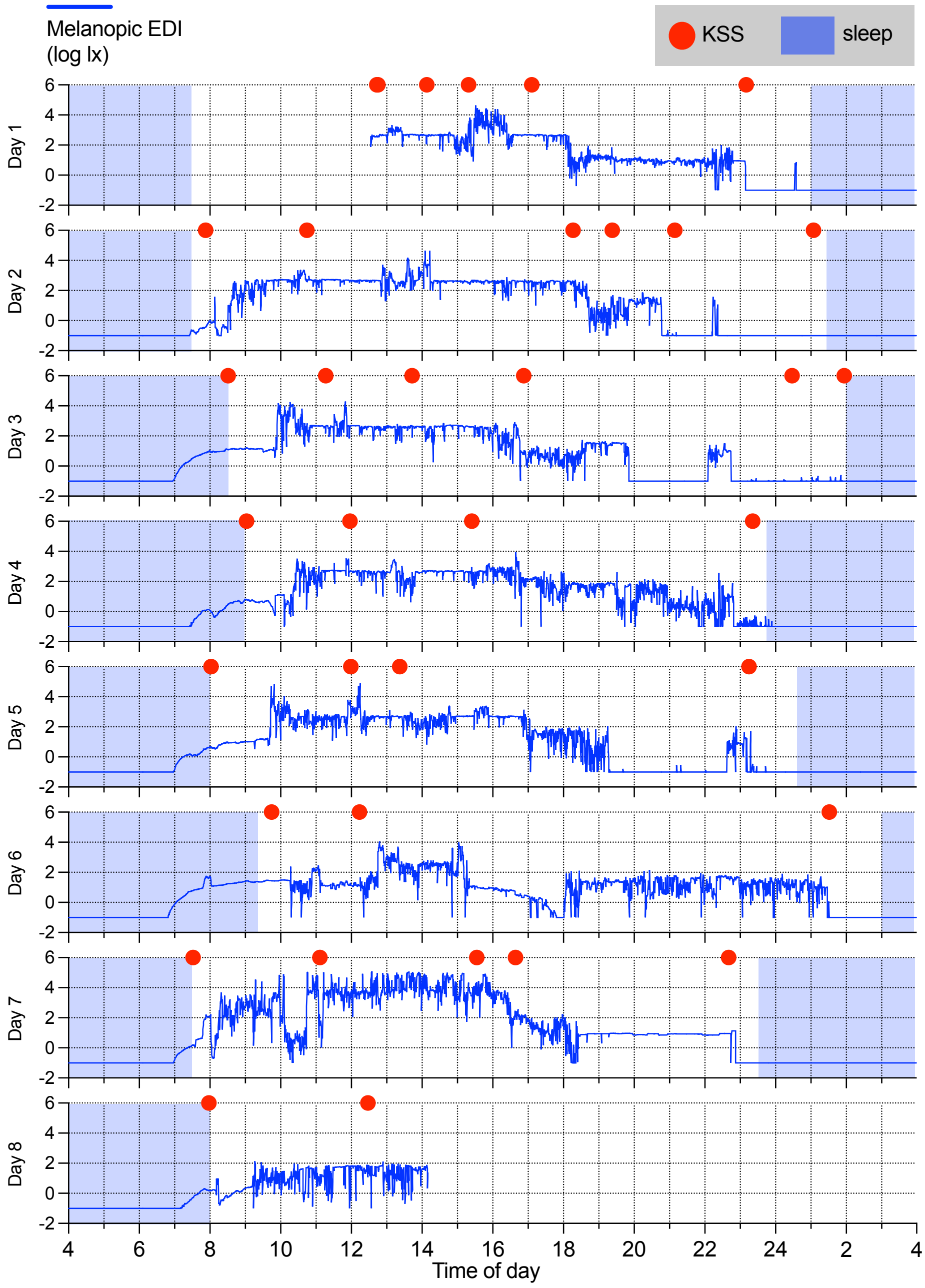

Figure S4A: Continuous melanopic equivalent daylight illuminance (EDI) measurements of a participant (blue line). The Y-axis shows melanopic EDI (log lx) and the X-axis shows time of day (clock time). The blue areas show subjectively reported sleep periods. The red circles show the times when sleepiness was recorded.

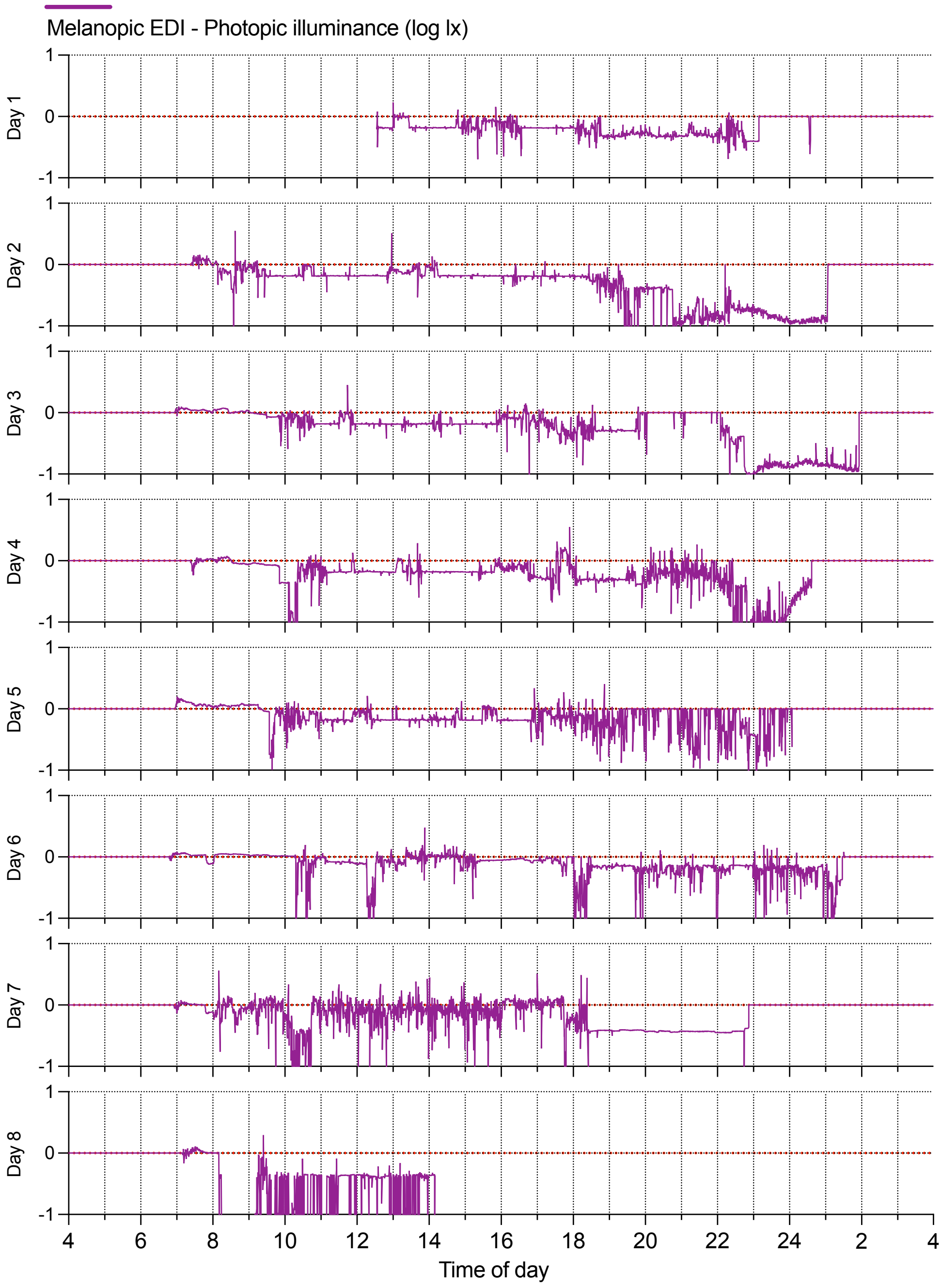

Figure S4B: Difference between melanopic EDI and photopic illuminance measurements of the same participant during the study period. The Y-axis shows the illuminance differences (log lx) and the X-axis shows time of day (clock time).

Associations between light exposure and sleep timing and sleepiness while awake in a sample of UK adults in everyday life

Supplementary materials

Table S1A: Sample description - Baseline

Baseline variables (N=59)	Sociodemographic and general health characteristics
Age	18 – 25 years (37%)<br>26 – 30 years (32%)<br>31 – 35 years (17%)<br>Other (between 36 – 60 years, 14%)
Sex	Male (34%)<br>Female (66%)
Employment status	full-time employed (34%)<br>Part-time employed (13%)<br>Student (49%)<br>Other (Unemployed or homemaker, 3%)
Shift work (occupation type)	Only 1 participant reported an occupation sometimes requiring night shift work
Education	Higher education (83%)
Subjective health	Poor (3%)<br>Fair (10%)<br>Good (56%)<br>Very good (31%)
Caffeine intake	2.5 units per day (range: 0 – 9)
Smoking	Never (88%)<br>1–5 (7%)<br>5–10 (3%)<br>Over 10 (2%)
Alcohol	Never (25%)<br>Special occasions only (32%)<br>1-2 in a week (31%)<br>3-4 in a week (12%)
Eye disorders	1 with color blindness and 1 with night blindness
Sleep disorders	3 with insomnia and 1 with sleep paralysis
Mental health disorders	12 with depression and/or anxiety, 1 eating disorder, 1 PTSD
Brain disorders	1 with functional neurological disorder
Sleep medications	None
Medications	4 with anti-depressants<br>3 with asthma inhalers<br>1 with high blood pressure & anti-arrhythmic<br>3 with nicotine-replacement
Subjective chronotype	Definitely a morning type (10%)<br>Rather more a morning type than an evening type (44%)<br>Rather more an evening type than a morning type (22%)<br>Definitely an evening type (24%)
Pittsburgh Sleep Quality Index (PSQI)	The median PSQI score was 4 (max: 11, mean: 4.8, SD: 2.3) and the most frequent sleep problem was higher sleep latency amongst participants
International Physical Activity Questionnaire (IPAQ)	High physical activity (51%)<br>Moderate physical activity (47%)<br>Low physical activity (2%)
Munich Chronotype Questionnaire (MCTQ)	Midsleep time on free days corrected for sleep debt on workdays (MCTQ MSFsc) was 4.7am with a normal distribution (SD=1.6; range: 0.6 – 8.3)<br>The mean social jetlag was 0.9h (SD=0.8)

Associations between light exposure and sleep timing and sleepiness while awake in a sample of UK adults in everyday life

Supplementary materials

Table S1B: Sample description - Daily sleep and work survey

Daily variables (N=478 days)	Sleep, work, and habits
Response per participant	range: 4-11 days per participant
Workdays	58% of records were from workdays (this included studying days for students), 65% of which included commuting to a work/study place
Going to work time	09:30 (SD=2.0)
Return from work time	18:26 (SD=2.8)
Workplaces	Home (35%)<br>Office (10%)<br>Outside (1%)<br>University (51%)<br>Other (3%)
Travel to workplaces	Walking (36%)<br>Bicycle (21%)<br>Car/motor vehicle (19%)<br>Public transport (22%)<br>Other (2%)
Shift work	Amongst all testing days, 7 days were reported to include shifts for 4 participants, but their sleep/wake schedules were comparable to the rest of the participants (Only 1 participant returned home from work after midnight)<br>Mean wake time 08:37 (Min: 06:40)<br>Mean bedtime 00:48 (Max: 06:00)<br>Mean sleep duration 6.75h (Min: 2.75h)<br>Mean going to work time 09:37 (between 07:30-18:00)<br>Mean return from work time 22:38 (between 18:00-05:55)
Screen duration (phone, display, TV)	<1hour (6%)<br>1-2hours (9%)<br>2-4hours (22%)<br>>4hours (63%)
Time spent outdoor	<1hour (32%)<br>1-2hours (39%)<br>2-4hours (19%)<br>>4hours (10%)
Caffeine intake	2.0 units per day (range: 0 – 8)
Alcohol consumption	23% of days (the median alcohol unit of 3)
Smoking behaviour	13% of days (in which 47% smoked between 1 and 5)
Napping	12% of days
Subjective sleep quality	Very bad (2%)<br>Bad (14%)<br>Fair (35%)<br>Good (36%)<br>Very good (13%)
Bedtime	00:19 (SD=2.0)
Wake time	08:22 (SD=1.9)
Sleep duration	7.3h (SD=1.3)
Sleep onset latency	0.4h (SD=0.5)
Sleep efficiency	90.5% (SD=10.3)

Associations between light exposure and sleep timing and sleepiness while awake in a sample of UK adults in everyday life

Supplementary materials

Table S2: Baseline sociodemographic and general health questionnaire

Questions	Answers
How old are you?	• Under 18<br>• 18 – 25<br>• 26 – 30<br>• 31 – 35<br>• 36 – 40<br>• 41 – 45<br>• 46 – 50<br>• 51 – 55<br>• 56 – 60<br>• 61 – 65<br>• Over 65
What is your sex?	• Male<br>• Female<br>• Prefer not to answer
What is your employment status? (if part-time or full-time employed) Write your occupation to the text box?	• Student<br>• Working part-time<br>• Working full-time<br>• Unemployed and looking for work<br>• A homemaker or stay-at-home parent<br>• Retired<br>• If other, please write below
Over the past 2 weeks, does your work involve shift work?	• Never or rarely<br>• Sometimes<br>• Always
Over the past 2 weeks, which shift pattern(s) did you follow for this job?	• Night<br>• Day<br>• Mixed
Do you have college or university degree?	• No<br>• Yes
What degree are you studying?	• Bachelor’s degree<br>• Master’s degree<br>• Doctoral degree
You may have heard of “morning” and “evening” types of people. Which of these do you consider yourself to be?	• Definitely a “morning” type<br>• Rather more a “morning” type than an “evening” type<br>• Rather more an “evening” type than a “morning” type<br>• Definitely an “evening” type
In a typical day, how frequently do you use cigarettes/electronic cigarettes/nicotine patch?	• Prefer not to answer<br>• Never<br>• 1 –5<br>• 5 – 10<br>• 10+
How much of the following caffeinated drinks do you consume in a typical week?	• Coffee……….. cups<br>• Tea (excluding decaf)……….. cups<br>• Energy drinks……….. cups
In a typical week, how often do you consume alcohol?	• Prefer not to answer<br>• Never<br>• Special occasions only<br>• Once or twice a week<br>• There or four times a week<br>• Daily or almost daily

How do you describe your general health?	• Very poor<br>• Poor<br>• Fair<br>• Good<br>• Very good
Has a doctor ever told you that you have eye disease? If yes, which type?	• No<br>• Colour blindness<br>• Glaucoma<br>• Retinal Degeneration<br>• Macular Degeneration<br>• Cataract<br>• Unsure<br>• Prefer not to answer<br>• If other, please write below
Has a doctor ever told you that you have a sleep disorder? If yes, which type?	• No<br>• Insomnia<br>• Sleep apnoea<br>• Restless leg syndrome<br>• Narcolepsy<br>• Unsure<br>• Prefer not to answer<br>• If other, please write below
Has a doctor ever told you that you have a mental health disorder? If yes, which type?	• No<br>• ADHD<br>• Depression<br>• Anxiety<br>• Schizophrenia<br>• Autism spectrum disorder<br>• Unsure<br>• Prefer not to answer<br>• If other, please write below
Has a doctor ever told you that you have a disorder of the brain/nerves? If yes, which type?	• No<br>• Stroke<br>• Parkinson’s<br>• Multiple Sclerosis<br>• Unsure<br>• Prefer not to answer<br>• If other, please write below
Do you take any of these medications frequently (at least 1/week)? If yes, which type?	• No<br>• For high blood pressure (such as beta-blockers)<br>• Nicotine-replacement products (including gum and nicotine-containing e-liquids)<br>• Anti-arrhythmic (for heart rhythm problems)<br>• Asthma inhalers<br>• Anti-depressants/anti-anxiety medication<br>• Sleeping tablets<br>• For ADD/ADHD (attention deficit disorder)<br>• Thyroid hormones<br>• Unsure<br>• Prefer not to answer<br>• If other, please write below

Associations between light exposure and sleep timing and sleepiness while awake in a sample of UK adults in everyday life

Supplementary materials

Table S3: Sleep and work diary

Questions	Answers
**The following questions are about 24 hours before you wake up today. (Please give times in 24-hour time)**	
Was yesterday a workday or free day?	• Workday<br>• Free day
What was your workplace yesterday?	• University<br>• Outside<br>• Office<br>• Store<br>• Warehouse<br>• Factory<br>• Vehicle<br>• Home<br>• If other, please write below
Did you commute to university/work yesterday?	• No<br>• Yes
How did you travel to/from university/work yesterday?	• Car/motor vehicle<br>• Public transport<br>• Bicycle<br>• Walking<br>• If other, please write below
Did your work involve shift work yesterday?	• No<br>• Yes
Did you go to work multiple times yesterday because of your shifts? 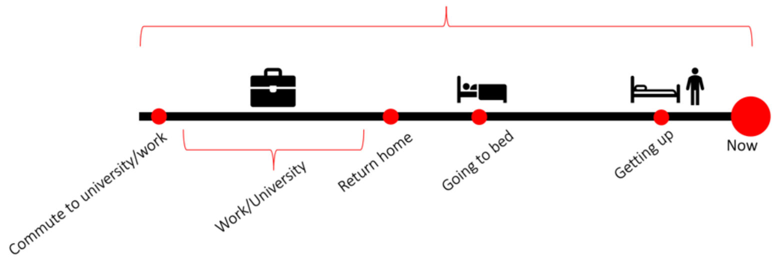 (Was your work and sleep schedule different than the schedule shown in the figure above) If you went to work multiple times yesterday because of your shifts, please write your work schedule below	• No<br>• Yes
When did you leave your home to commute to university/work yesterday?	
What time did you return home yesterday?	
**Please tick your level of agreement with the statements listed below:**	
Overall, the light was comfortable at the place where you study/work yesterday.	• Strongly disagree<br>• Somewhat disagree<br>• Neither agree nor disagree<br>• Somewhat agree<br>• Strongly agree
The lighting was well distributed at the place where you study/work yesterday.	• Strongly disagree<br>• Somewhat disagree<br>• Neither agree nor disagree<br>• Somewhat agree<br>• Strongly agree
The brightness of light was enough to work/study correctly yesterday.	• Strongly disagree<br>• Somewhat disagree<br>• Neither agree nor disagree

	• Somewhat agree<br>• Strongly agree
The colour of light was adequate at the place where you study/work yesterday.	• Strongly disagree<br>• Somewhat disagree<br>• Neither agree nor disagree<br>• Somewhat agree<br>• Strongly agree
How was the colour of lighting at the place where you study/work yesterday?	• Cool colours (bluish white)<br>• Neutral white<br>• Warm colours (yellowish white through red)
On average, how much time did you spend looking at computer/mobile phone/tablet display yesterday?	• <1 hour<br>• 1 – 2 hours<br>• 2 – 4 hours<br>• >4 hours
How much time did you spend outdoor in daylight yesterday (e.g. walking, running, bicycle, sitting outside)?	• <1 hour<br>• 1 – 2 hours<br>• 2 – 4 hours<br>• >4 hours
How much of the following caffeinated drinks did you consume yesterday?	• Coffee……(cups)<br>• Tea……(cup excluding caffeine-free)<br>• Energy drinks ……(cans)
How frequently did you use cigarettes/electronic cigarettes/nicotine patch yesterday?	• Prefer not to answer<br>• Never<br>• 1 – 5<br>• 5 – 10<br>• 10+
Did you consume alcohol yesterday? If yes, how many units of alcohol did you consume yesterday? (1 unit = half a pint of beer, small glass of wine, shot of spirit)	• Prefer not to answer<br>• No<br>• Yes (please write below) ……..
What time did you go to bed yesterday?	
How many minutes after going to bed did you fall asleep yesterday?	
What time did you wake up today?	
How many hours of actual sleep did you get yesterday?	
Have you had any naps yesterday?	• No<br>• Yes
How would you rate your sleep quality yesterday?	• Very bad<br>• Bad<br>• Fair<br>• Good<br>• Very good

Associations between light exposure and sleep timing and sleepiness while awake in a sample of UK adults in everyday life

Supplementary materials

Table S4: Repeated sleepiness survey

Questions	Answers
Using the scale below; how awake or sleepy do you feel right now?	1. Extremely alert<br>2. Very alert<br>3. Alert<br>4. Rather alert<br>5. Neither alert nor sleepy<br>6. Some signs of sleepiness<br>7. Sleepy, but no effort to keep awake<br>8. Sleepy, but with some effort to keep awake<br>9. Very sleepy, great effort to keep awake, fighting sleep<br>10. Extremely sleepy, can't keep awake
Have you slept since your last entry?	• No<br>• Yes
(If yes) When did you fall asleep?	
(If yes) When did you wake up?	
